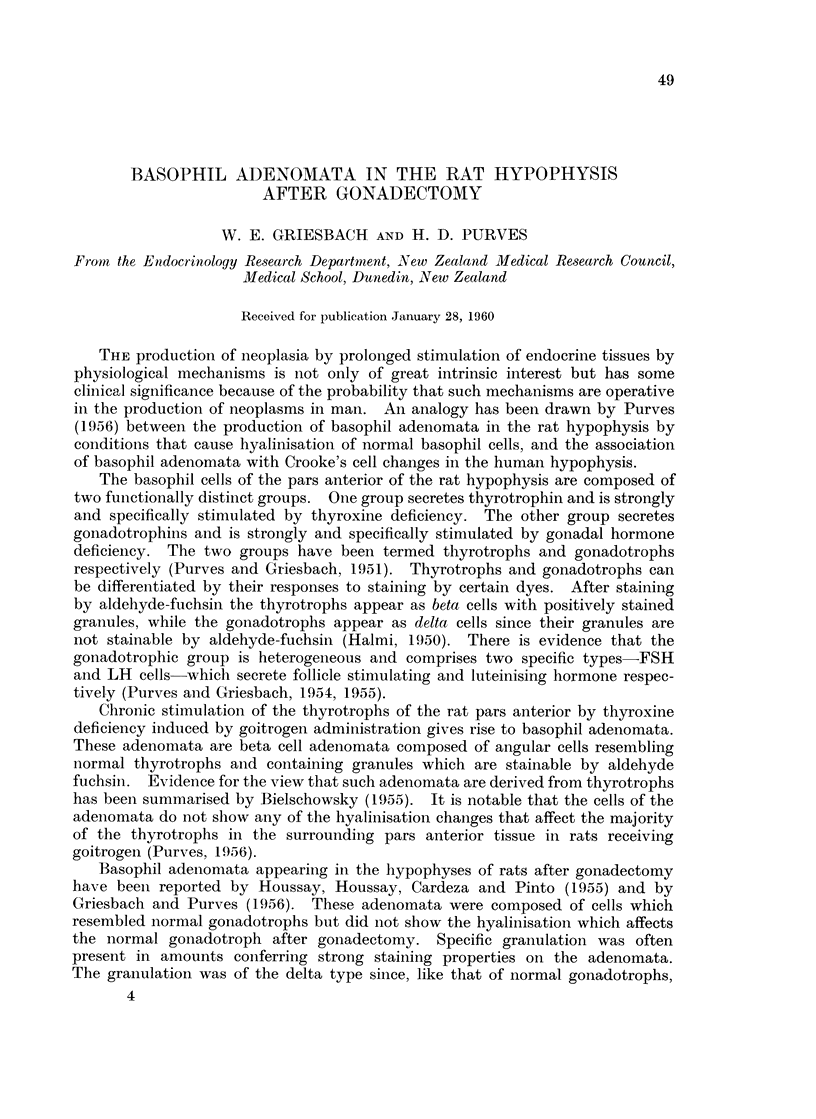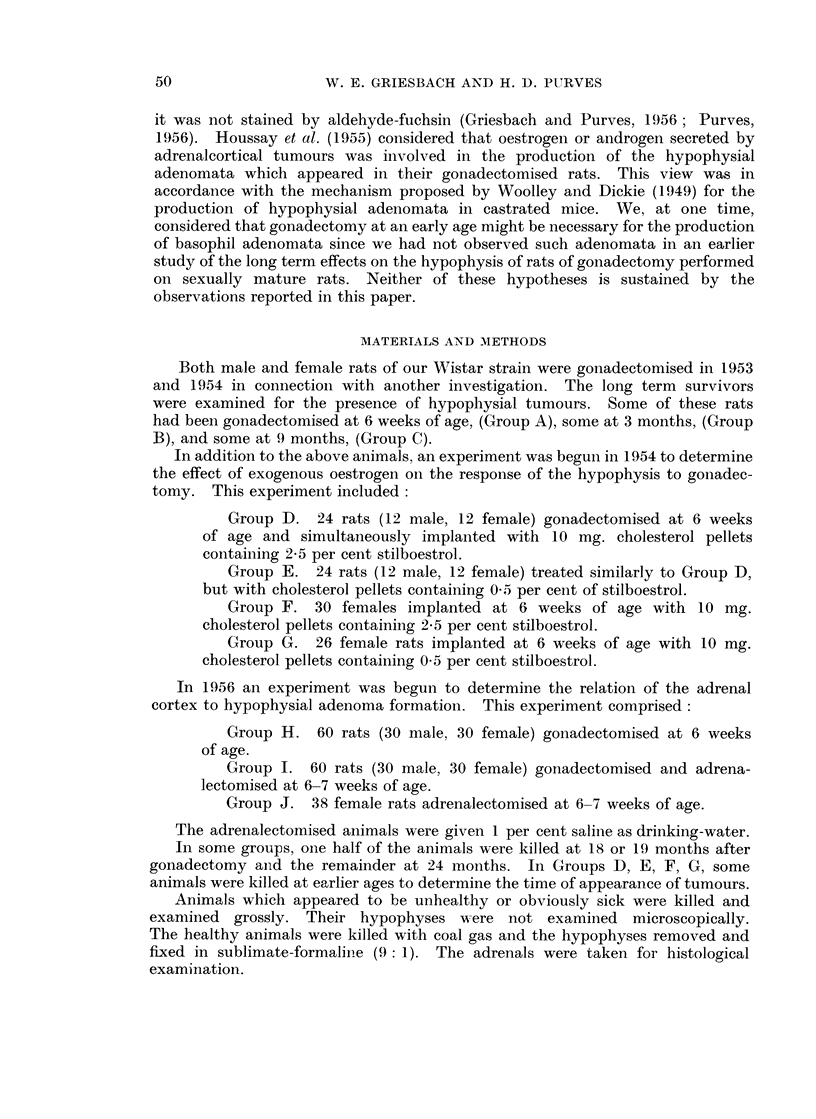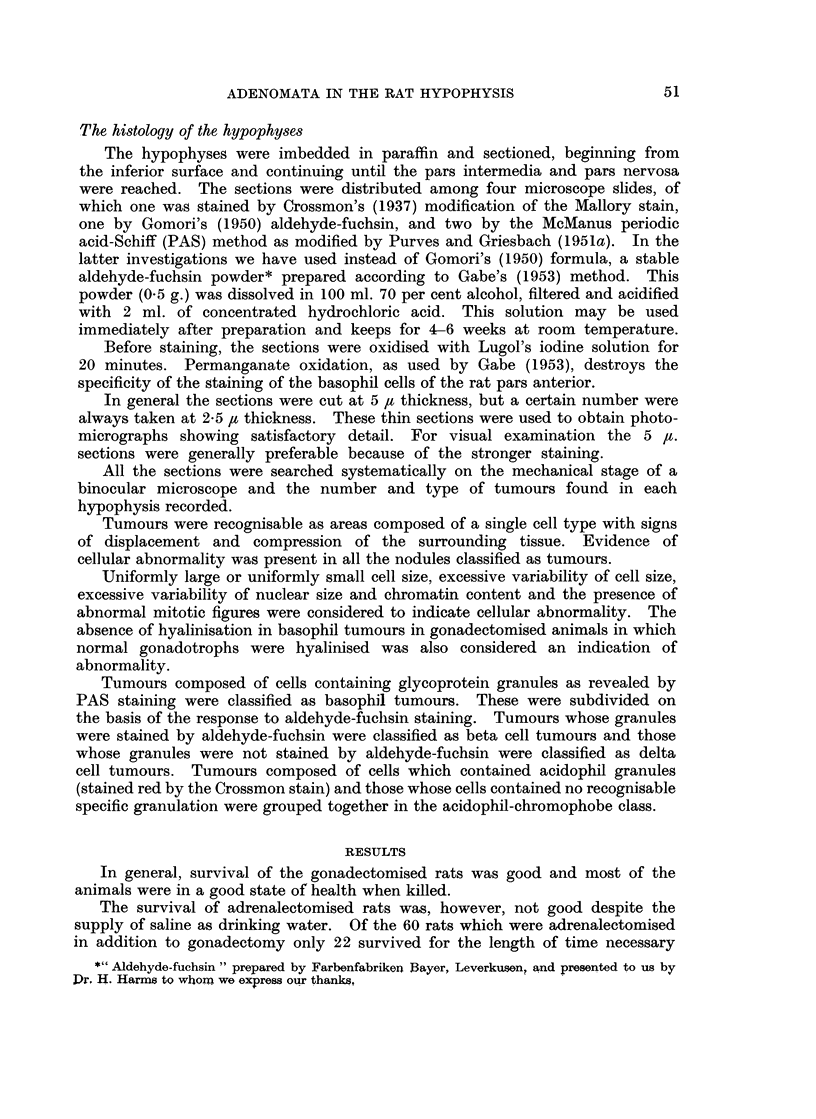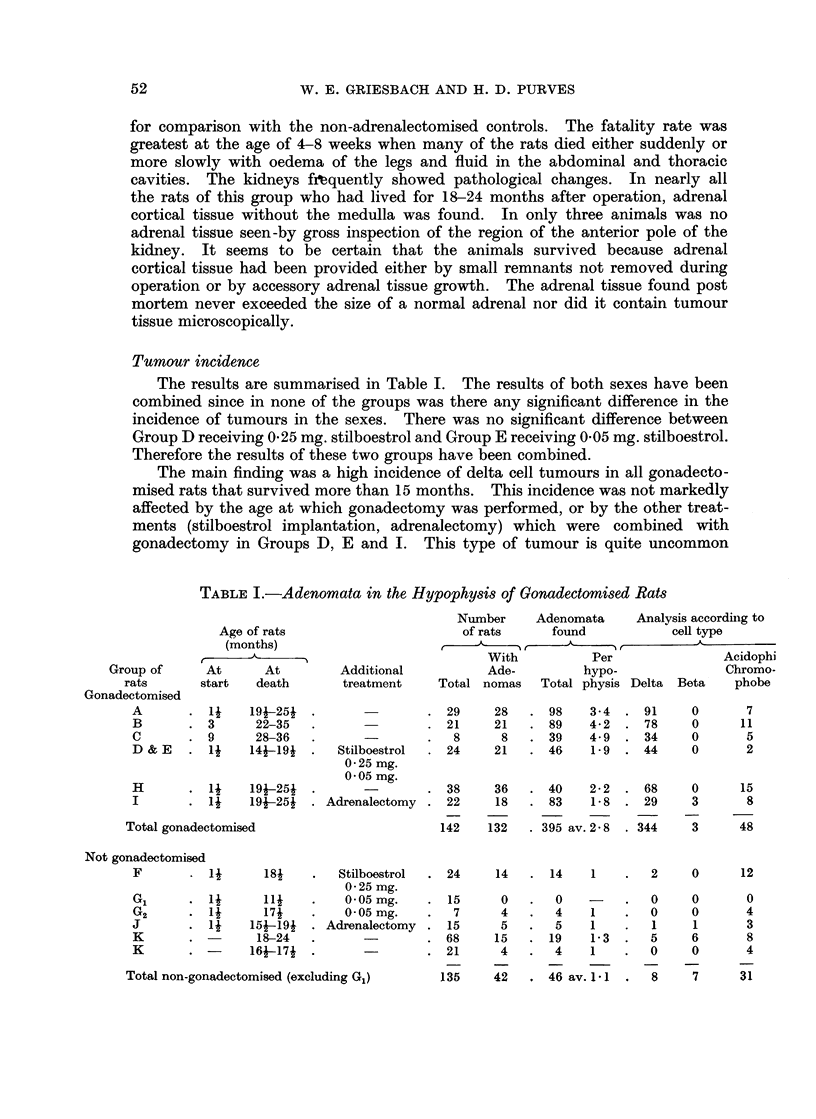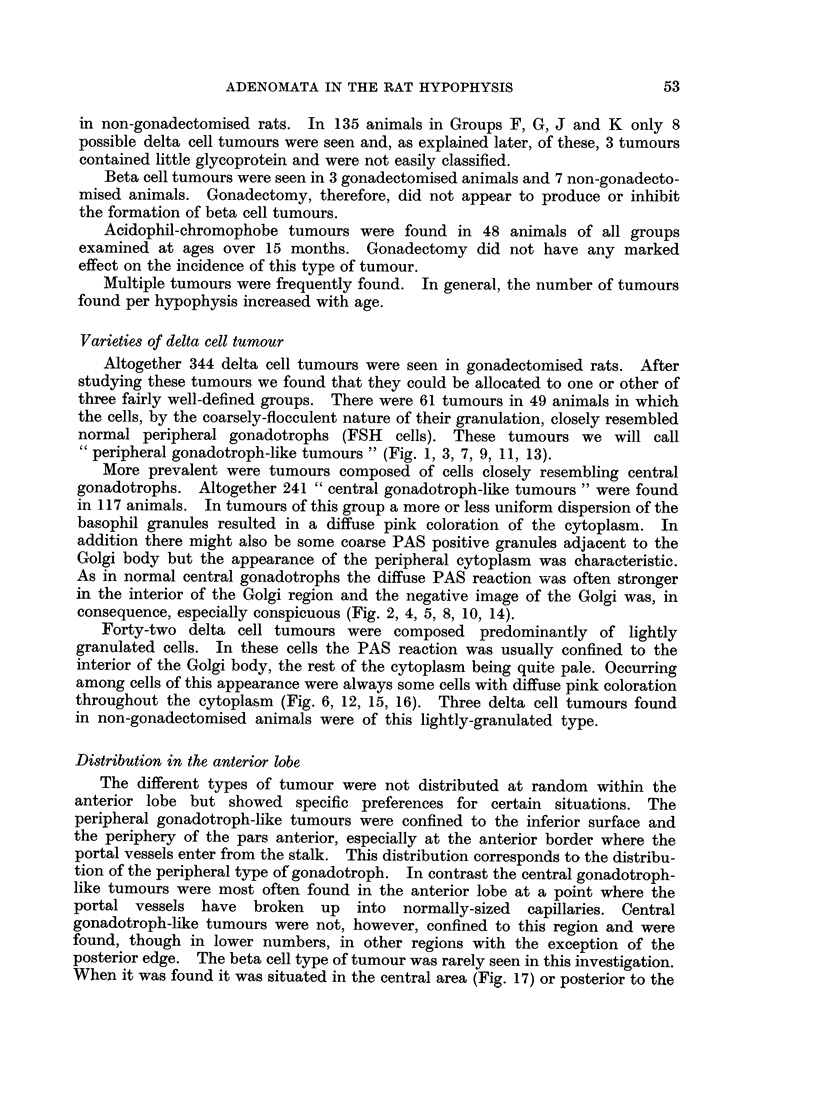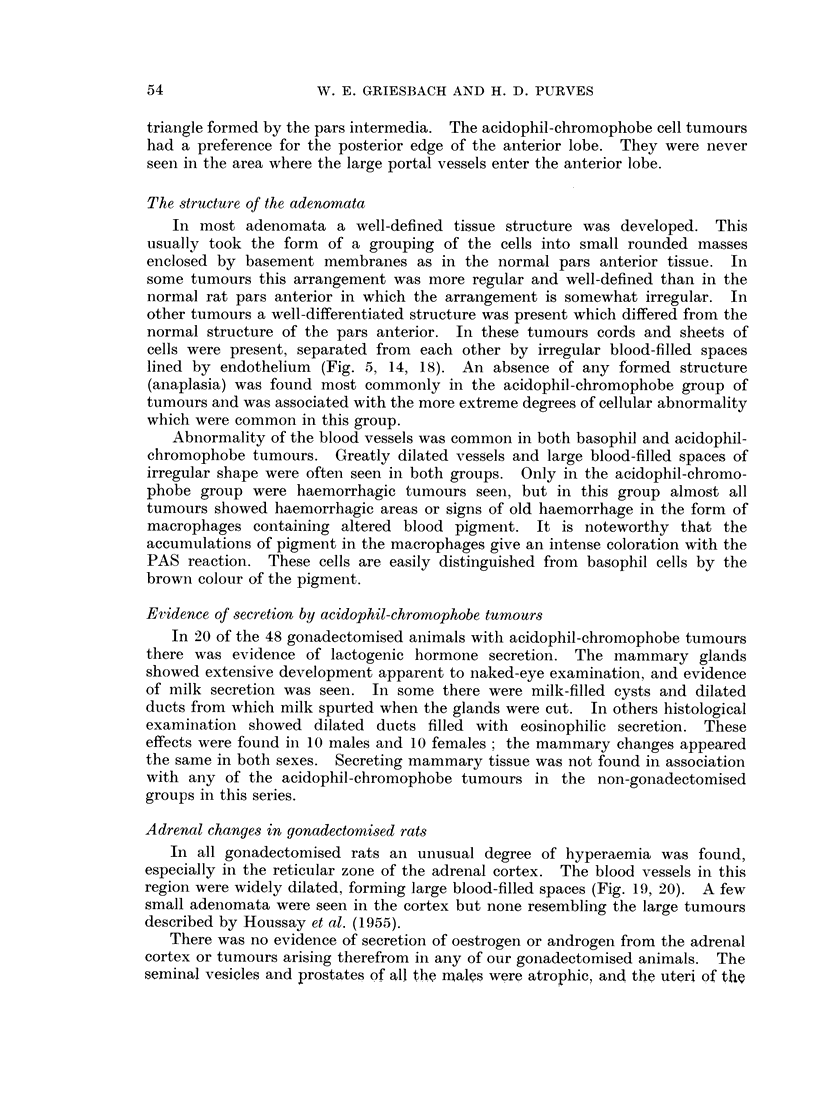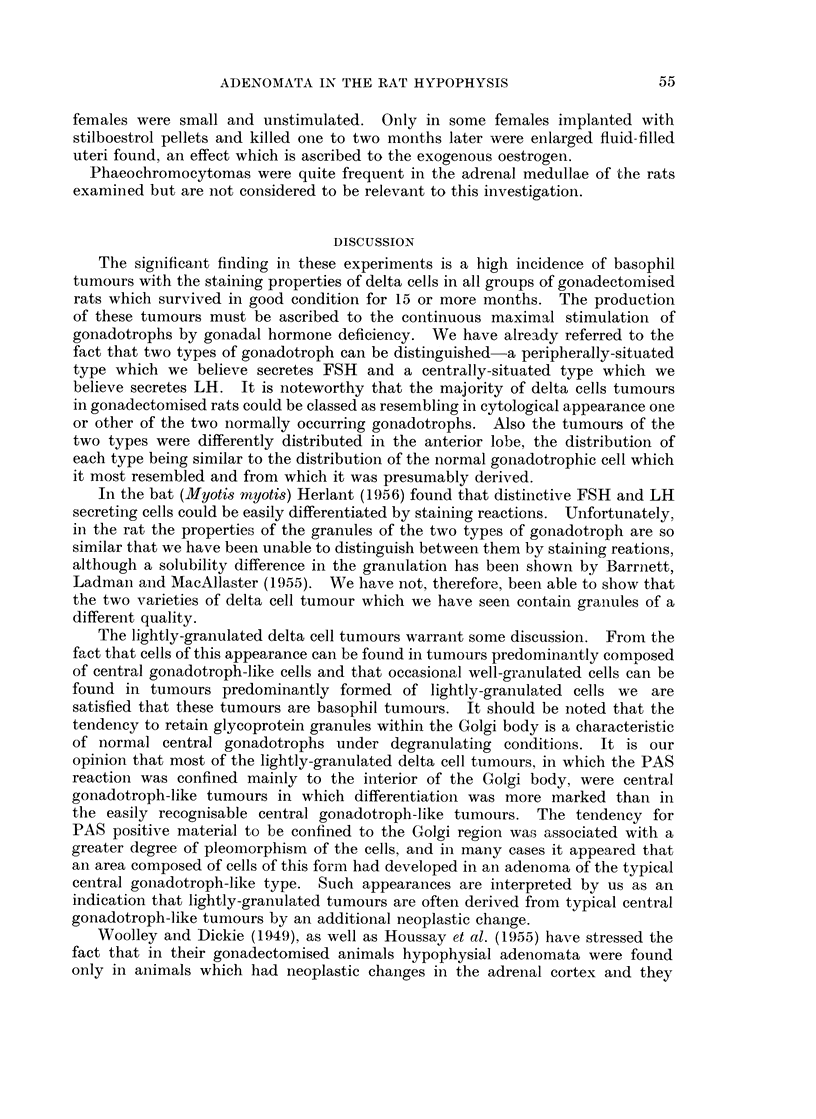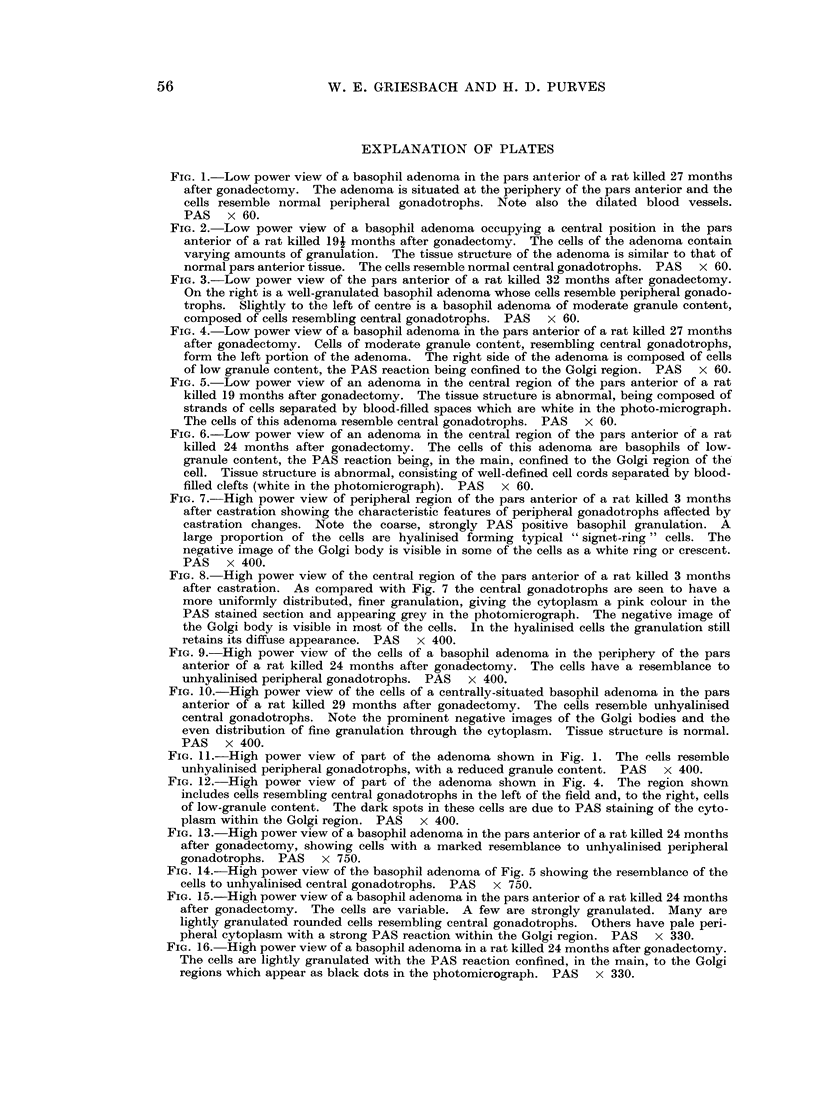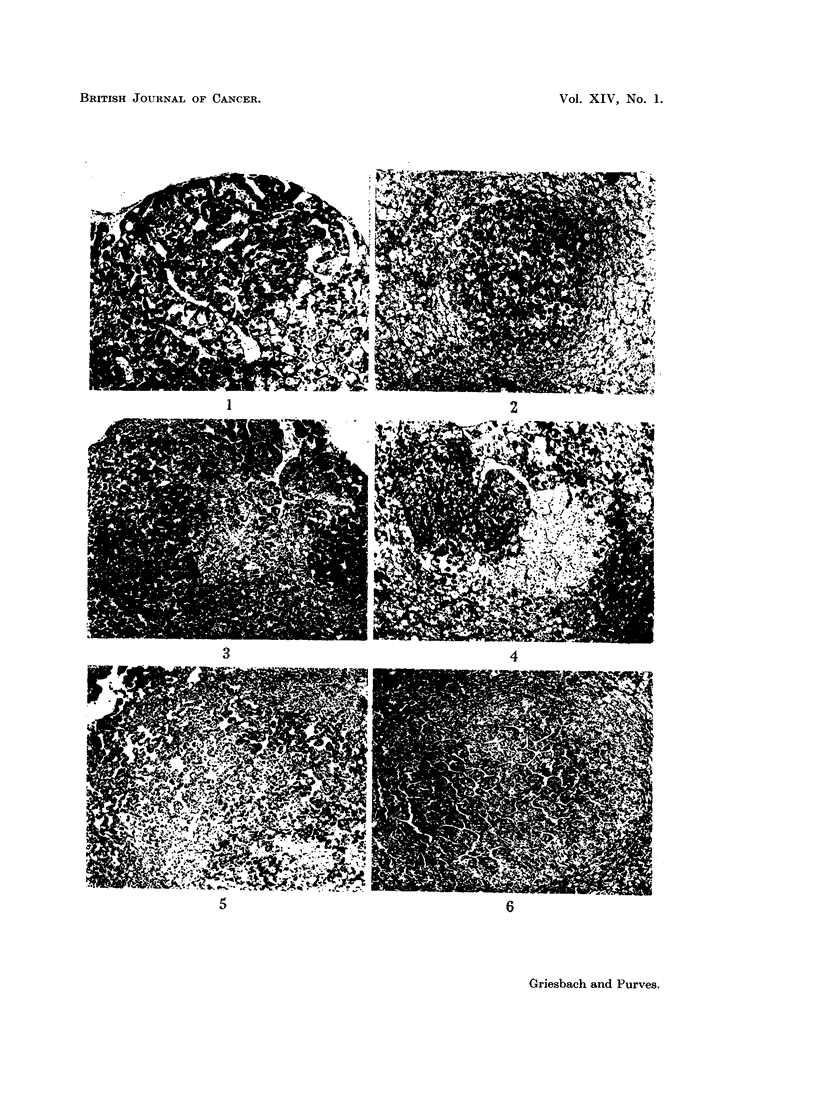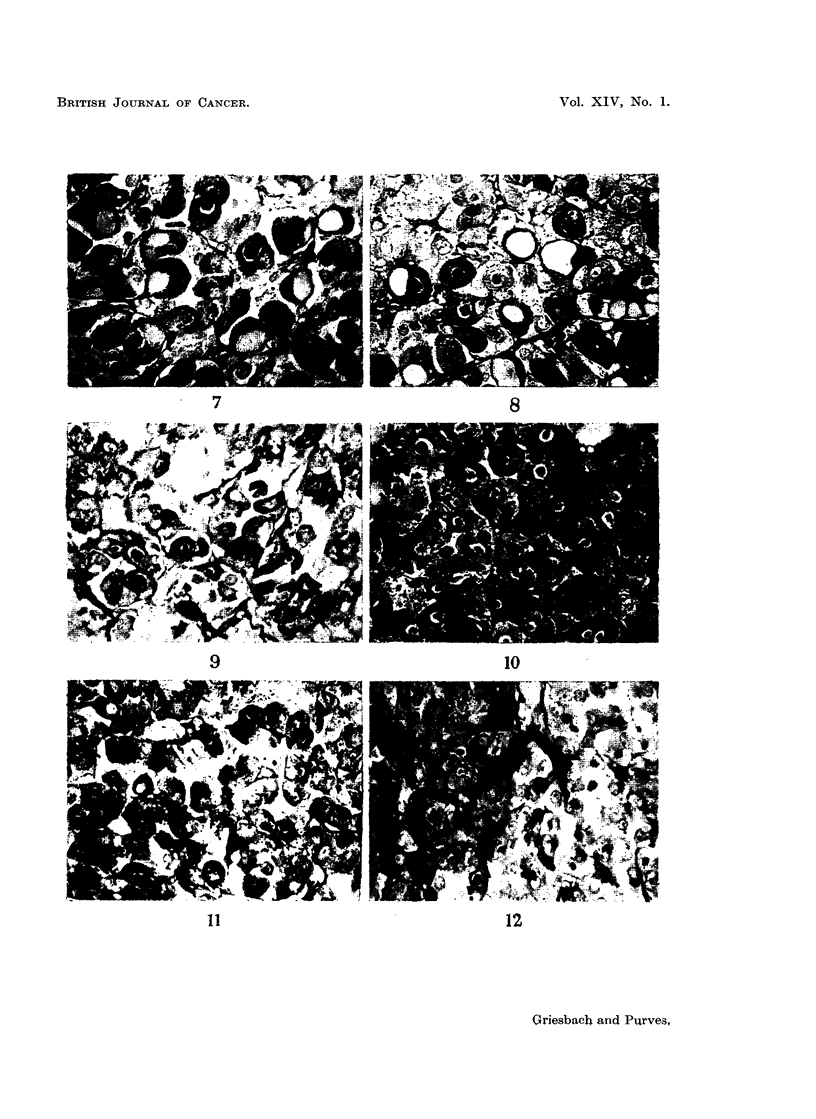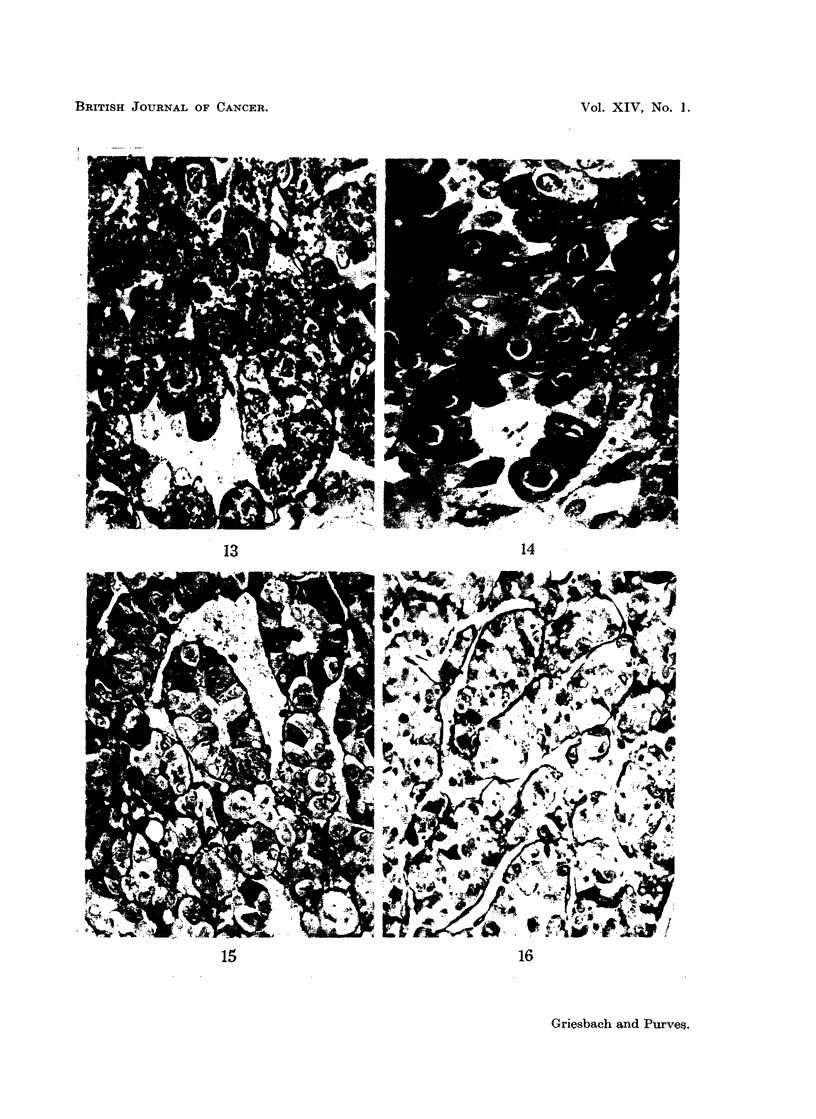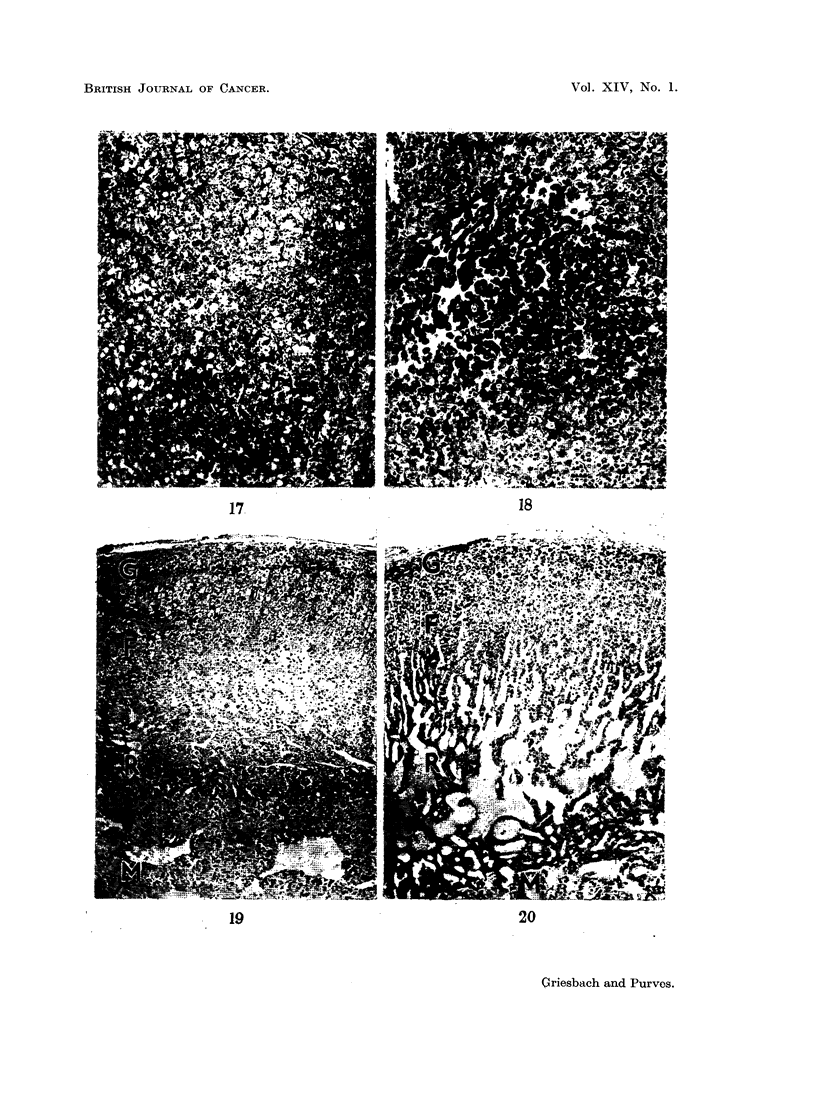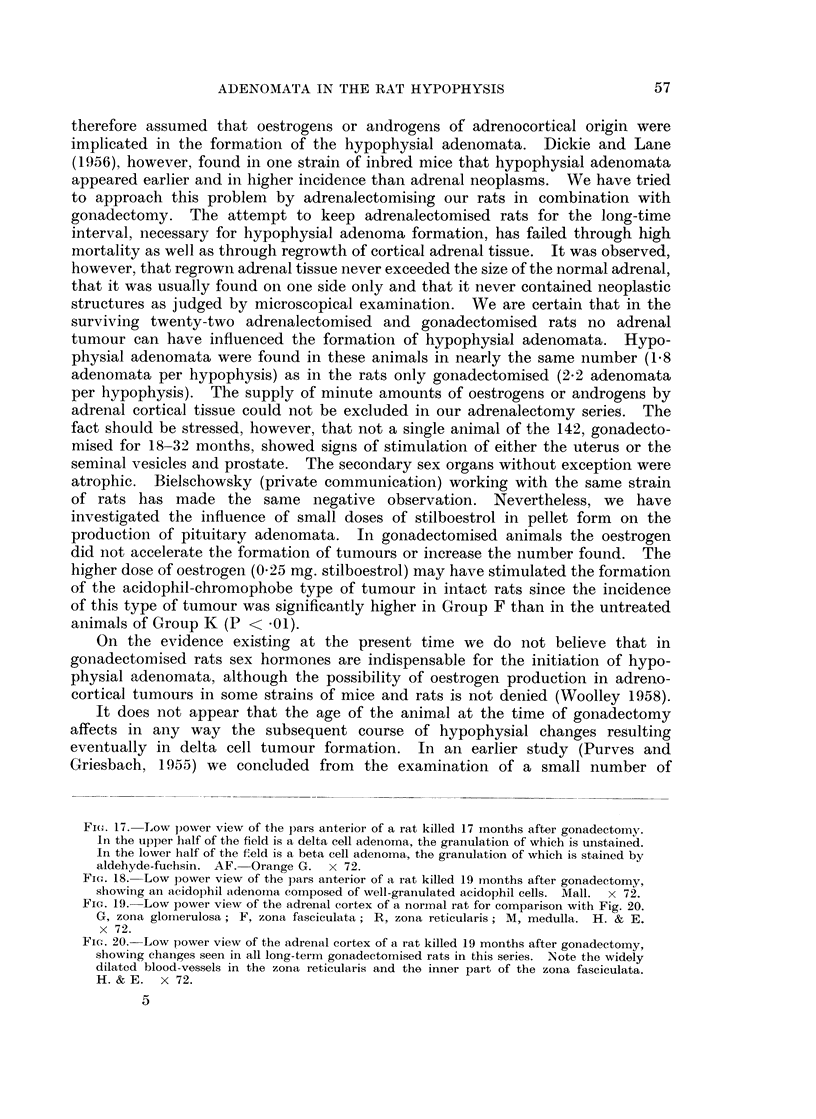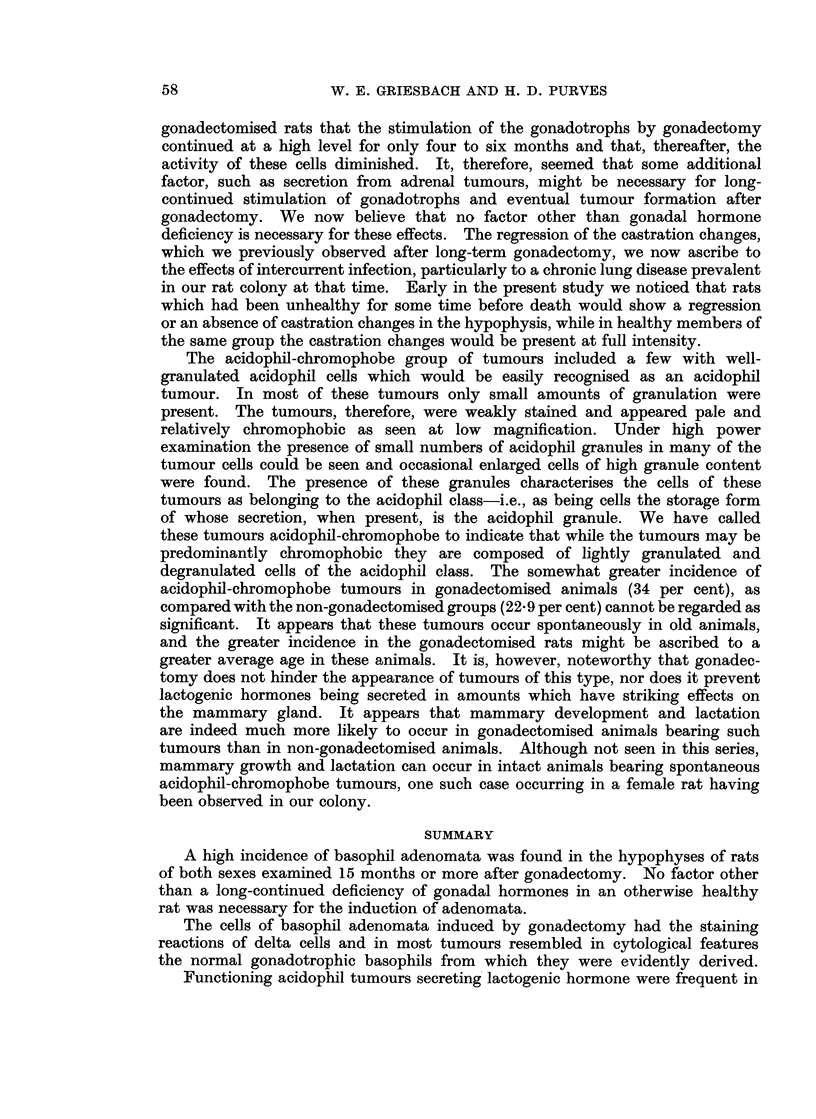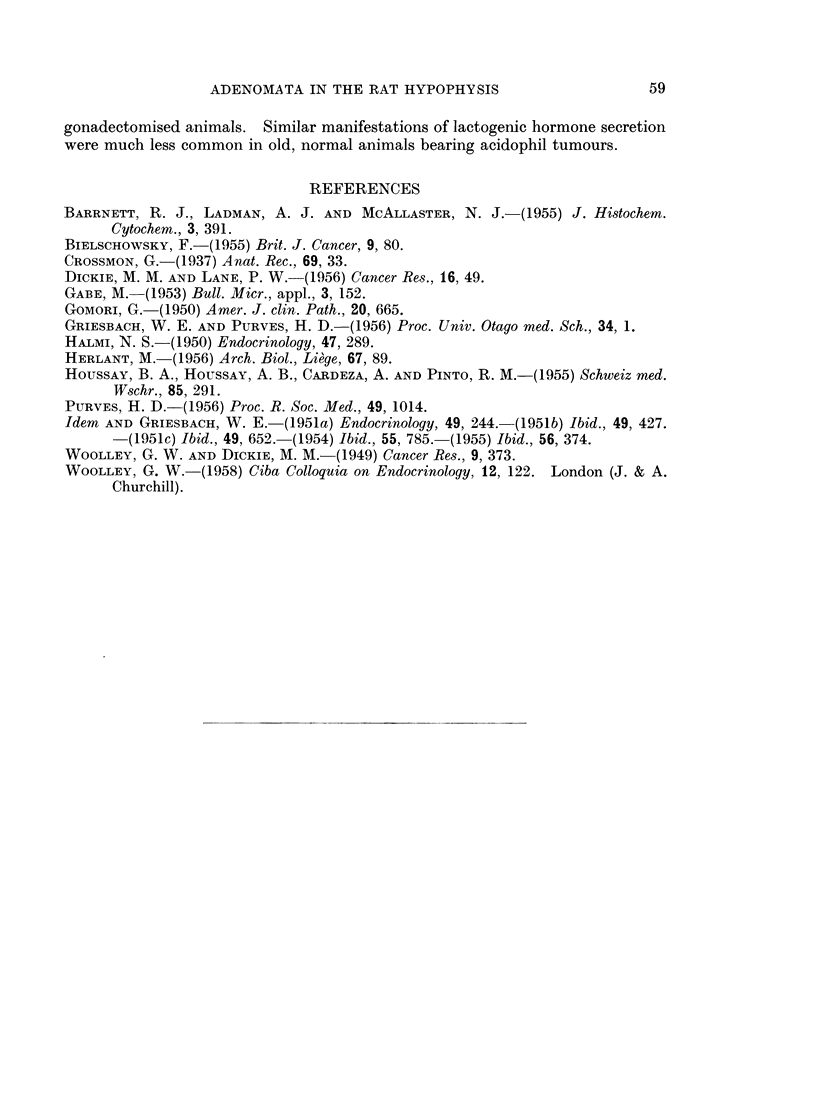# Basophil Adenomata in the Rat Hypophysis After Gonadectomy

**DOI:** 10.1038/bjc.1960.6

**Published:** 1960-03

**Authors:** W. E. Griesbach, H. D. Purves

## Abstract

**Images:**


### BASOPHIL ADENOMATA IN THE RAT HYPOPHYSIS AFTER GONADECTOMY

W. E. GRIESBACH AND H. D. PURVES

*From the Endocrinology Research Department, New Zealand Medical Research Council, Medical School, Dunedin, New Zealand*

Received for publication January 28, 1960

THE production of neoplasia by prolonged stimulation of endocrine tissues by physiological mechanisms is not only of great intrinsic interest but has some clinical significance because of the probability that such mechanisms are operative in the production of neoplasms in man. An analogy has been drawn by Purves (1956) between the production of basophil adenomata in the rat hypophysis by conditions that cause hyalinisation of normal basophil cells, and the association of basophil adenomata with Crooke's cell changes in the human hypophysis.

The basophil cells of the pars anterior of the rat hypophysis are composed of two functionally distinct groups. One group secretes thyrotrophin and is strongly and specifically stimulated by thyroxine deficiency. The other group secretes gonadotrophins and is strongly and specifically stimulated by gonadal hormone deficiency. The two groups have been termed thyrotrophs and gonadotrophs respectively (Purves and Griesbach, 1951). Thyrotrophs and gonadotrophs can be differentiated by their responses to staining by certain dyes. After staining by aldehyde-fuchsin the thyrotrophs appear as *beta* cells with positively stained granules, while the gonadotrophs appear as *delta* cells since their granules are not stainable by aldehyde-fuchsin (Halmi, 1950). There is evidence that the gonadotrophic group is heterogeneous and comprises two specific types—FSH and LH cells—which secrete follicle stimulating and luteinising hormone respectively (Purves and Griesbach, 1954, 1955).

Chronic stimulation of the thyrotrophs of the rat pars anterior by thyroxine deficiency induced by goitrogen administration gives rise to basophil adenomata. These adenomata are beta cell adenomata composed of angular cells resembling normal thyrotrophs and containing granules which are stainable by aldehyde fuchsin. Evidence for the view that such adenomata are derived from thyrotrophs has been summarised by Bielschowsky (1955). It is notable that the cells of the adenomata do not show any of the hyalinisation changes that affect the majority of the thyrotrophs in the surrounding pars anterior tissue in rats receiving goitrogen (Purves, 1956).

Basophil adenomata appearing in the hypophyses of rats after gonadectomy have been reported by Houssay, Houssay, Cardeza and Pinto (1955) and by Griesbach and Purves (1956). These adenomata were composed of cells which resembled normal gonadotrophs but did not show the hyalinisation which affects the normal gonadotroph after gonadectomy. Specific granulation was often present in amounts conferring strong staining properties on the adenomata. The granulation was of the delta type since, like that of normal gonadotrophs, it was not stained by aldehyde-fuchsin (Griesbach and Purves, 1956; Purves, 1956). Houssay *et al.* (1955) considered that oestrogen or androgen secreted by adrenalcortical tumours was involved in the production of the hypophysial adenomata which appeared in their gonadectomised rats. This view was in accordance with the mechanism proposed by Woolley and Dickie (1949) for the production of hypophysial adenomata in castrated mice. We, at one time, considered that gonadectomy at an early age might be necessary for the production of basophil adenomata since we had not observed such adenomata in an earlier study of the long term effects on the hypophysis of rats of gonadectomy performed on sexually mature rats. Neither of these hypotheses is sustained by the observations reported in this paper.

### MATERIALS AND METHODS

Both male and female rats of our Wistar strain were gonadectomised in 1953 and 1954 in connection with another investigation. The long term survivors were examined for the presence of hypophysial tumours. Some of these rats had been gonadectomised at 6 weeks of age, (Group A), some at 3 months, (Group B), and some at 9 months, (Group C).

In addition to the above animals, an experiment was begun in 1954 to determine the effect of exogenous oestrogen on the response of the hypophysis to gonadectomy. This experiment included:

Group D. 24 rats (12 male, 12 female) gonadectomised at 6 weeks of age and simultaneously implanted with 10 mg. cholesterol pellets containing 2·5 per cent stilboestrol.

Group E. 24 rats (12 male, 12 female) treated similarly to Group D, but with cholesterol pellets containing 0·5 per cent of stilboestrol.

Group F. 30 females implanted at 6 weeks of age with 10 mg. cholesterol pellets containing 2·5 per cent stilboestrol.

Group G. 26 female rats implanted at 6 weeks of age with 10 mg. cholesterol pellets containing 0·5 per cent stilboestrol.

In 1956 an experiment was begun to determine the relation of the adrenal cortex to hypophysial adenoma formation. This experiment comprised:

Group H. 60 rats (30 male, 30 female) gonadectomised at 6 weeks of age.

Group I. 60 rats (30 male, 30 female) gonadectomised and adrenalectomised at 6–7 weeks of age.

Group J. 38 female rats adrenalectomised at 6–7 weeks of age.

The adrenalectomised animals were given 1 per cent saline as drinking-water.

In some groups, one half of the animals were killed at 18 or 19 months after gonadectomy and the remainder at 24 months. In Groups D, E, F, G, some animals were killed at earlier ages to determine the time of appearance of tumours.

Animals which appeared to be unhealthy or obviously sick were killed and examined grossly. Their hypophyses were not examined microscopically. The healthy animals were killed with coal gas and the hypophyses removed and fixed in sublimate-formaline (9 : 1). The adrenals were taken for histological examination.

*The histology of the hypophyses*

The hypophyses were imbedded in paraffin and sectioned, beginning from the inferior surface and continuing until the pars intermedia and pars nervosa were reached. The sections were distributed among four microscope slides, of which one was stained by Crossmon's (1937) modification of the Mallory stain, one by Gomori's (1950) aldehyde-fuchsin, and two by the McManus periodic acid-Schiff (PAS) method as modified by Purves and Griesbach (1951*a*). In the latter investigations we have used instead of Gomori's (1950) formula, a stable aldehyde-fuchsin powder* prepared according to Gabe's (1953) method. This powder (0·5 g.) was dissolved in 100 ml. 70 per cent alcohol, filtered and acidified with 2 ml. of concentrated hydrochloric acid. This solution may be used immediately after preparation and keeps for 4–6 weeks at room temperature.

Before staining, the sections were oxidised with Lugol's iodine solution for 20 minutes. Permanganate oxidation, as used by Gabe (1953), destroys the specificity of the staining of the basophil cells of the rat pars anterior.

In general the sections were cut at 5 $\mu$ thickness, but a certain number were always taken at 2·5 $\mu$ thickness. These thin sections were used to obtain photomicrographs showing satisfactory detail. For visual examination the 5 $\mu$. sections were generally preferable because of the stronger staining.

All the sections were searched systematically on the mechanical stage of a binocular microscope and the number and type of tumours found in each hypophysis recorded.

Tumours were recognisable as areas composed of a single cell type with signs of displacement and compression of the surrounding tissue. Evidence of cellular abnormality was present in all the nodules classified as tumours.

Uniformly large or uniformly small cell size, excessive variability of cell size, excessive variability of nuclear size and chromatin content and the presence of abnormal mitotic figures were considered to indicate cellular abnormality. The absence of hyalinisation in basophil tumours in gonadectomised animals in which normal gonadotrophs were hyalinised was also considered an indication of abnormality.

Tumours composed of cells containing glycoprotein granules as revealed by PAS staining were classified as basophil tumours. These were subdivided on the basis of the response to aldehyde-fuchsin staining. Tumours whose granules were stained by aldehyde-fuchsin were classified as beta cell tumours and those whose granules were not stained by aldehyde-fuchsin were classified as delta cell tumours. Tumours composed of cells which contained acidophil granules (stained red by the Crossmon stain) and those whose cells contained no recognisable specific granulation were grouped together in the acidophil-chromophobe class.

### RESULTS

In general, survival of the gonadectomised rats was good and most of the animals were in a good state of health when killed.

The survival of adrenalectomised rats was, however, not good despite the supply of saline as drinking water. Of the 60 rats which were adrenalectomised in addition to gonadectomy only 22 survived for the length of time necessary

*" Aldehyde-fuchsin " prepared by Farbenfabriken Bayer, Leverkusen, and presented to us by Dr. H. Harms to whom we express our thanks.

for comparison with the non-adrenalectomised controls. The fatality rate was greatest at the age of 4–8 weeks when many of the rats died either suddenly or more slowly with oedema of the legs and fluid in the abdominal and thoracic cavities. The kidneys frequently showed pathological changes. In nearly all the rats of this group who had lived for 18–24 months after operation, adrenal cortical tissue without the medulla was found. In only three animals was no adrenal tissue seen by gross inspection of the region of the anterior pole of the kidney. It seems to be certain that the animals survived because adrenal cortical tissue had been provided either by small remnants not removed during operation or by accessory adrenal tissue growth. The adrenal tissue found post mortem never exceeded the size of a normal adrenal nor did it contain tumour tissue microscopically.

*Tumour incidence*

The results are summarised in Table I. The results of both sexes have been combined since in none of the groups was there any significant difference in the incidence of tumours in the sexes. There was no significant difference between Group D receiving 0·25 mg. stilboestrol and Group E receiving 0·05 mg. stilboestrol. Therefore the results of these two groups have been combined.

The main finding was a high incidence of delta cell tumours in all gonadectomised rats that survived more than 15 months. This incidence was not markedly affected by the age at which gonadectomy was performed, or by the other treatments (stilboestrol implantation, adrenalectomy) which were combined with gonadectomy in Groups D, E and I. This type of tumour is quite uncommon

TABLE I.—*Adenomata in the Hypophysis of Gonadectomised Rats*

| Group of rats | Age of rats (months) | | Additional treatment | Number of rats | | Adenomata found | | Analysis according to cell type | | |
|---|---|---|---|---|---|---|---|---|---|---|
| | At start | At death | | Total | With Adenomas | Total | Per hypophysis | Delta | Beta | Acidophi Chromophobe |
| Gonadectomised | | | | | | | | | | |
| A | 1½ | 19½–25½ | — | 29 | 28 | 98 | 3·4 | 91 | 0 | 7 |
| B | 3 | 22–35 | — | 21 | 21 | 89 | 4·2 | 78 | 0 | 11 |
| C | 9 | 28–36 | — | 8 | 8 | 39 | 4·9 | 34 | 0 | 5 |
| D & E | 1½ | 14½–19½ | Stilboestrol 0·25 mg. 0·05 mg. | 24 | 21 | 46 | 1·9 | 44 | 0 | 2 |
| H | 1½ | 19½–25½ | — | 38 | 36 | 40 | 2·2 | 68 | 0 | 15 |
| I | 1½ | 19½–25½ | Adrenalectomy | 22 | 18 | 83 | 1·8 | 29 | 3 | 8 |
| Total gonadectomised | | | | 142 | 132 | 395 | av. 2·8 | 344 | 3 | 48 |
| Not gonadectomised | | | | | | | | | | |
| F | 1½ | 18½ | Stilboestrol 0·25 mg. | 24 | 14 | 14 | 1 | 2 | 0 | 12 |
| $G_1$ | 1½ | 11½ | 0·05 mg. | 15 | 0 | 0 | — | 0 | 0 | 0 |
| $G_2$ | 1½ | 17½ | 0·05 mg. | 7 | 4 | 4 | 1 | 0 | 0 | 4 |
| J | 1½ | 15½–19½ | Adrenalectomy | 15 | 5 | 5 | 1 | 1 | 1 | 3 |
| K | — | 18–24 | — | 68 | 15 | 19 | 1·3 | 5 | 6 | 8 |
| K | — | 16½–17½ | — | 21 | 4 | 4 | 1 | 0 | 0 | 4 |
| Total non-gonadectomised (excluding $G_1$) | | | | 135 | 42 | 46 | av. 1·1 | 8 | 7 | 31 |

in non-gonadectomised rats. In 135 animals in Groups F, G, J and K only 8 possible delta cell tumours were seen and, as explained later, of these, 3 tumours contained little glycoprotein and were not easily classified.

Beta cell tumours were seen in 3 gonadectomised animals and 7 non-gonadectomised animals. Gonadectomy, therefore, did not appear to produce or inhibit the formation of beta cell tumours.

Acidophil-chromophobe tumours were found in 48 animals of all groups examined at ages over 15 months. Gonadectomy did not have any marked effect on the incidence of this type of tumour.

Multiple tumours were frequently found. In general, the number of tumours found per hypophysis increased with age.

*Varieties of delta cell tumour*

Altogether 344 delta cell tumours were seen in gonadectomised rats. After studying these tumours we found that they could be allocated to one or other of three fairly well-defined groups. There were 61 tumours in 49 animals in which the cells, by the coarsely-flocculent nature of their granulation, closely resembled normal peripheral gonadotrophs (FSH cells). These tumours we will call " peripheral gonadotroph-like tumours " (Fig. 1, 3, 7, 9, 11, 13).

More prevalent were tumours composed of cells closely resembling central gonadotrophs. Altogether 241 " central gonadotroph-like tumours " were found in 117 animals. In tumours of this group a more or less uniform dispersion of the basophil granules resulted in a diffuse pink coloration of the cytoplasm. In addition there might also be some coarse PAS positive granules adjacent to the Golgi body but the appearance of the peripheral cytoplasm was characteristic. As in normal central gonadotrophs the diffuse PAS reaction was often stronger in the interior of the Golgi region and the negative image of the Golgi was, in consequence, especially conspicuous (Fig. 2, 4, 5, 8, 10, 14).

Forty-two delta cell tumours were composed predominantly of lightly granulated cells. In these cells the PAS reaction was usually confined to the interior of the Golgi body, the rest of the cytoplasm being quite pale. Occurring among cells of this appearance were always some cells with diffuse pink coloration throughout the cytoplasm (Fig. 6, 12, 15, 16). Three delta cell tumours found in non-gonadectomised animals were of this lightly-granulated type.

*Distribution in the anterior lobe*

The different types of tumour were not distributed at random within the anterior lobe but showed specific preferences for certain situations. The peripheral gonadotroph-like tumours were confined to the inferior surface and the periphery of the pars anterior, especially at the anterior border where the portal vessels enter from the stalk. This distribution corresponds to the distribution of the peripheral type of gonadotroph. In contrast the central gonadotroph-like tumours were most often found in the anterior lobe at a point where the portal vessels have broken up into normally-sized capillaries. Central gonadotroph-like tumours were not, however, confined to this region and were found, though in lower numbers, in other regions with the exception of the posterior edge. The beta cell type of tumour was rarely seen in this investigation. When it was found it was situated in the central area (Fig. 17) or posterior to the triangle formed by the pars intermedia. The acidophil-chromophobe cell tumours had a preference for the posterior edge of the anterior lobe. They were never seen in the area where the large portal vessels enter the anterior lobe.

*The structure of the adenomata*

In most adenomata a well-defined tissue structure was developed. This usually took the form of a grouping of the cells into small rounded masses enclosed by basement membranes as in the normal pars anterior tissue. In some tumours this arrangement was more regular and well-defined than in the normal rat pars anterior in which the arrangement is somewhat irregular. In other tumours a well-differentiated structure was present which differed from the normal structure of the pars anterior. In these tumours cords and sheets of cells were present, separated from each other by irregular blood-filled spaces lined by endothelium (Fig. 5, 14, 18). An absence of any formed structure (anaplasia) was found most commonly in the acidophil-chromophobe group of tumours and was associated with the more extreme degrees of cellular abnormality which were common in this group.

Abnormality of the blood vessels was common in both basophil and acidophil-chromophobe tumours. Greatly dilated vessels and large blood-filled spaces of irregular shape were often seen in both groups. Only in the acidophil-chromophobe group were haemorrhagic tumours seen, but in this group almost all tumours showed haemorrhagic areas or signs of old haemorrhage in the form of macrophages containing altered blood pigment. It is noteworthy that the accumulations of pigment in the macrophages give an intense coloration with the PAS reaction. These cells are easily distinguished from basophil cells by the brown colour of the pigment.

*Evidence of secretion by acidophil-chromophobe tumours*

In 20 of the 48 gonadectomised animals with acidophil-chromophobe tumours there was evidence of lactogenic hormone secretion. The mammary glands showed extensive development apparent to naked-eye examination, and evidence of milk secretion was seen. In some there were milk-filled cysts and dilated ducts from which milk spurted when the glands were cut. In others histological examination showed dilated ducts filled with eosinophilic secretion. These effects were found in 10 males and 10 females ; the mammary changes appeared the same in both sexes. Secreting mammary tissue was not found in association with any of the acidophil-chromophobe tumours in the non-gonadectomised groups in this series.

*Adrenal changes in gonadectomised rats*

In all gonadectomised rats an unusual degree of hyperaemia was found, especially in the reticular zone of the adrenal cortex. The blood vessels in this region were widely dilated, forming large blood-filled spaces (Fig. 19, 20). A few small adenomata were seen in the cortex but none resembling the large tumours described by Houssay *et al.* (1955).

There was no evidence of secretion of oestrogen or androgen from the adrenal cortex or tumours arising therefrom in any of our gonadectomised animals. The seminal vesicles and prostates of all the males were atrophic, and the uteri of the females were small and unstimulated. Only in some females implanted with stilboestrol pellets and killed one to two months later were enlarged fluid-filled uteri found, an effect which is ascribed to the exogenous oestrogen.

Phaeochromocytomas were quite frequent in the adrenal medullae of the rats examined but are not considered to be relevant to this investigation.

### DISCUSSION

The significant finding in these experiments is a high incidence of basophil tumours with the staining properties of delta cells in all groups of gonadectomised rats which survived in good condition for 15 or more months. The production of these tumours must be ascribed to the continuous maximal stimulation of gonadotrophs by gonadal hormone deficiency. We have already referred to the fact that two types of gonadotroph can be distinguished—a peripherally-situated type which we believe secretes FSH and a centrally-situated type which we believe secretes LH. It is noteworthy that the majority of delta cells tumours in gonadectomised rats could be classed as resembling in cytological appearance one or other of the two normally occurring gonadotrophs. Also the tumours of the two types were differently distributed in the anterior lobe, the distribution of each type being similar to the distribution of the normal gonadotrophic cell which it most resembled and from which it was presumably derived.

In the bat (*Myotis myotis*) Herlant (1956) found that distinctive FSH and LH secreting cells could be easily differentiated by staining reactions. Unfortunately, in the rat the properties of the granules of the two types of gonadotroph are so similar that we have been unable to distinguish between them by staining reations, although a solubility difference in the granulation has been shown by Barrnett, Ladman and MacAllaster (1955). We have not, therefore, been able to show that the two varieties of delta cell tumour which we have seen contain granules of a different quality.

The lightly-granulated delta cell tumours warrant some discussion. From the fact that cells of this appearance can be found in tumours predominantly composed of central gonadotroph-like cells and that occasional well-granulated cells can be found in tumours predominantly formed of lightly-granulated cells we are satisfied that these tumours are basophil tumours. It should be noted that the tendency to retain glycoprotein granules within the Golgi body is a characteristic of normal central gonadotrophs under degranulating conditions. It is our opinion that most of the lightly-granulated delta cell tumours, in which the PAS reaction was confined mainly to the interior of the Golgi body, were central gonadotroph-like tumours in which differentiation was more marked than in the easily recognisable central gonadotroph-like tumours. The tendency for PAS positive material to be confined to the Golgi region was associated with a greater degree of pleomorphism of the cells, and in many cases it appeared that an area composed of cells of this form had developed in an adenoma of the typical central gonadotroph-like type. Such appearances are interpreted by us as an indication that lightly-granulated tumours are often derived from typical central gonadotroph-like tumours by an additional neoplastic change.

Woolley and Dickie (1949), as well as Houssay *et al.* (1955) have stressed the fact that in their gonadectomised animals hypophysial adenomata were found only in animals which had neoplastic changes in the adrenal cortex and they

### EXPLANATION OF PLATES

FIG. 1.—Low power view of a basophil adenoma in the pars anterior of a rat killed 27 months after gonadectomy. The adenoma is situated at the periphery of the pars anterior and the cells resemble normal peripheral gonadotrophs. Note also the dilated blood vessels. PAS × 60.

FIG. 2.—Low power view of a basophil adenoma occupying a central position in the pars anterior of a rat killed 19½ months after gonadectomy. The cells of the adenoma contain varying amounts of granulation. The tissue structure of the adenoma is similar to that of normal pars anterior tissue. The cells resemble normal central gonadotrophs. PAS × 60.

FIG. 3.—Low power view of the pars anterior of a rat killed 32 months after gonadectomy. On the right is a well-granulated basophil adenoma whose cells resemble peripheral gonadotrophs. Slightly to the left of centre is a basophil adenoma of moderate granule content, composed of cells resembling central gonadotrophs. PAS × 60.

FIG. 4.—Low power view of a basophil adenoma in the pars anterior of a rat killed 27 months after gonadectomy. Cells of moderate granule content, resembling central gonadotrophs, form the left portion of the adenoma. The right side of the adenoma is composed of cells of low granule content, the PAS reaction being confined to the Golgi region. PAS × 60.

FIG. 5.—Low power view of an adenoma in the central region of the pars anterior of a rat killed 19 months after gonadectomy. The tissue structure is abnormal, being composed of strands of cells separated by blood-filled spaces which are white in the photo-micrograph. The cells of this adenoma resemble central gonadotrophs. PAS × 60.

FIG. 6.—Low power view of an adenoma in the central region of the pars anterior of a rat killed 24 months after gonadectomy. The cells of this adenoma are basophils of low-granule content, the PAS reaction being, in the main, confined to the Golgi region of the cell. Tissue structure is abnormal, consisting of well-defined cell cords separated by blood-filled clefts (white in the photomicrograph). PAS × 60.

FIG. 7.—High power view of peripheral region of the pars anterior of a rat killed 3 months after castration showing the characteristic features of peripheral gonadotrophs affected by castration changes. Note the coarse, strongly PAS positive basophil granulation. A large proportion of the cells are hyalinised forming typical "signet-ring" cells. The negative image of the Golgi body is visible in some of the cells as a white ring or crescent. PAS × 400.

FIG. 8.—High power view of the central region of the pars anterior of a rat killed 3 months after castration. As compared with Fig. 7 the central gonadotrophs are seen to have a more uniformly distributed, finer granulation, giving the cytoplasm a pink colour in the PAS stained section and appearing grey in the photomicrograph. The negative image of the Golgi body is visible in most of the cells. In the hyalinised cells the granulation still retains its diffuse appearance. PAS × 400.

FIG. 9.—High power view of the cells of a basophil adenoma in the periphery of the pars anterior of a rat killed 24 months after gonadectomy. The cells have a resemblance to unhyalinised peripheral gonadotrophs. PAS × 400.

FIG. 10.—High power view of the cells of a centrally-situated basophil adenoma in the pars anterior of a rat killed 29 months after gonadectomy. The cells resemble unhyalinised central gonadotrophs. Note the prominent negative images of the Golgi bodies and the even distribution of fine granulation through the cytoplasm. Tissue structure is normal. PAS × 400.

FIG. 11.—High power view of part of the adenoma shown in Fig. 1. The cells resemble unhyalinised peripheral gonadotrophs, with a reduced granule content. PAS × 400.

FIG. 12.—High power view of part of the adenoma shown in Fig. 4. The region shown includes cells resembling central gonadotrophs in the left of the field and, to the right, cells of low-granule content. The dark spots in these cells are due to PAS staining of the cytoplasm within the Golgi region. PAS × 400.

FIG. 13.—High power view of a basophil adenoma in the pars anterior of a rat killed 24 months after gonadectomy, showing cells with a marked resemblance to unhyalinised peripheral gonadotrophs. PAS × 750.

FIG. 14.—High power view of the basophil adenoma of Fig. 5 showing the resemblance of the cells to unhyalinised central gonadotrophs. PAS × 750.

FIG. 15.—High power view of a basophil adenoma in the pars anterior of a rat killed 24 months after gonadectomy. The cells are variable. A few are strongly granulated. Many are lightly granulated rounded cells resembling central gonadotrophs. Others have pale peripheral cytoplasm with a strong PAS reaction within the Golgi region. PAS × 330.

FIG. 16.—High power view of a basophil adenoma in a rat killed 24 months after gonadectomy. The cells are lightly granulated with the PAS reaction confined, in the main, to the Golgi regions which appear as black dots in the photomicrograph. PAS × 330.

1

2

3

4

5

6

Griesbach and Purves.

7

8

9

10

11

12

Griesbach and Purves.

13

14

15

16

Griesbach and Purves.

17

18

19

20

Griesbach and Purves.

therefore assumed that oestrogens or androgens of adrenocortical origin were implicated in the formation of the hypophysial adenomata. Dickie and Lane (1956), however, found in one strain of inbred mice that hypophysial adenomata appeared earlier and in higher incidence than adrenal neoplasms. We have tried to approach this problem by adrenalectomising our rats in combination with gonadectomy. The attempt to keep adrenalectomised rats for the long-time interval, necessary for hypophysial adenoma formation, has failed through high mortality as well as through regrowth of cortical adrenal tissue. It was observed, however, that regrown adrenal tissue never exceeded the size of the normal adrenal, that it was usually found on one side only and that it never contained neoplastic structures as judged by microscopical examination. We are certain that in the surviving twenty-two adrenalectomised and gonadectomised rats no adrenal tumour can have influenced the formation of hypophysial adenomata. Hypophysial adenomata were found in these animals in nearly the same number (1·8 adenomata per hypophysis) as in the rats only gonadectomised (2·2 adenomata per hypophysis). The supply of minute amounts of oestrogens or androgens by adrenal cortical tissue could not be excluded in our adrenalectomy series. The fact should be stressed, however, that not a single animal of the 142, gonadectomised for 18–32 months, showed signs of stimulation of either the uterus or the seminal vesicles and prostate. The secondary sex organs without exception were atrophic. Bielschowsky (private communication) working with the same strain of rats has made the same negative observation. Nevertheless, we have investigated the influence of small doses of stilboestrol in pellet form on the production of pituitary adenomata. In gonadectomised animals the oestrogen did not accelerate the formation of tumours or increase the number found. The higher dose of oestrogen (0·25 mg. stilboestrol) may have stimulated the formation of the acidophil-chromophobe type of tumour in intact rats since the incidence of this type of tumour was significantly higher in Group F than in the untreated animals of Group K ($P < \cdot 01$).

On the evidence existing at the present time we do not believe that in gonadectomised rats sex hormones are indispensable for the initiation of hypophysial adenomata, although the possibility of oestrogen production in adrenocortical tumours in some strains of mice and rats is not denied (Woolley 1958).

It does not appear that the age of the animal at the time of gonadectomy affects in any way the subsequent course of hypophysial changes resulting eventually in delta cell tumour formation. In an earlier study (Purves and Griesbach, 1955) we concluded from the examination of a small number of

---

Fig. 17.—Low power view of the pars anterior of a rat killed 17 months after gonadectomy. In the upper half of the field is a delta cell adenoma, the granulation of which is unstained. In the lower half of the field is a beta cell adenoma, the granulation of which is stained by aldehyde-fuchsin. AF.—Orange G. × 72.

Fig. 18.—Low power view of the pars anterior of a rat killed 19 months after gonadectomy, showing an acidophil adenoma composed of well-granulated acidophil cells. Mall. × 72.

Fig. 19.—Low power view of the adrenal cortex of a normal rat for comparison with Fig. 20. G, zona glomerulosa ; F, zona fasciculata ; R, zona reticularis ; M, medulla. H. & E. × 72.

Fig. 20.—Low power view of the adrenal cortex of a rat killed 19 months after gonadectomy, showing changes seen in all long-term gonadectomised rats in this series. Note the widely dilated blood-vessels in the zona reticularis and the inner part of the zona fasciculata. H. & E. × 72.

gonadectomised rats that the stimulation of the gonadotrophs by gonadectomy continued at a high level for only four to six months and that, thereafter, the activity of these cells diminished. It, therefore, seemed that some additional factor, such as secretion from adrenal tumours, might be necessary for long-continued stimulation of gonadotrophs and eventual tumour formation after gonadectomy. We now believe that no factor other than gonadal hormone deficiency is necessary for these effects. The regression of the castration changes, which we previously observed after long-term gonadectomy, we now ascribe to the effects of intercurrent infection, particularly to a chronic lung disease prevalent in our rat colony at that time. Early in the present study we noticed that rats which had been unhealthy for some time before death would show a regression or an absence of castration changes in the hypophysis, while in healthy members of the same group the castration changes would be present at full intensity.

The acidophil-chromophobe group of tumours included a few with well-granulated acidophil cells which would be easily recognised as an acidophil tumour. In most of these tumours only small amounts of granulation were present. The tumours, therefore, were weakly stained and appeared pale and relatively chromophobic as seen at low magnification. Under high power examination the presence of small numbers of acidophil granules in many of the tumour cells could be seen and occasional enlarged cells of high granule content were found. The presence of these granules characterises the cells of these tumours as belonging to the acidophil class—i.e., as being cells the storage form of whose secretion, when present, is the acidophil granule. We have called these tumours acidophil-chromophobe to indicate that while the tumours may be predominantly chromophobic they are composed of lightly granulated and degranulated cells of the acidophil class. The somewhat greater incidence of acidophil-chromophobe tumours in gonadectomised animals (34 per cent), as compared with the non-gonadectomised groups (22·9 per cent) cannot be regarded as significant. It appears that these tumours occur spontaneously in old animals, and the greater incidence in the gonadectomised rats might be ascribed to a greater average age in these animals. It is, however, noteworthy that gonadectomy does not hinder the appearance of tumours of this type, nor does it prevent lactogenic hormones being secreted in amounts which have striking effects on the mammary gland. It appears that mammary development and lactation are indeed much more likely to occur in gonadectomised animals bearing such tumours than in non-gonadectomised animals. Although not seen in this series, mammary growth and lactation can occur in intact animals bearing spontaneous acidophil-chromophobe tumours, one such case occurring in a female rat having been observed in our colony.

### SUMMARY

A high incidence of basophil adenomata was found in the hypophyses of rats of both sexes examined 15 months or more after gonadectomy. No factor other than a long-continued deficiency of gonadal hormones in an otherwise healthy rat was necessary for the induction of adenomata.

The cells of basophil adenomata induced by gonadectomy had the staining reactions of delta cells and in most tumours resembled in cytological features the normal gonadotrophic basophils from which they were evidently derived.

Functioning acidophil tumours secreting lactogenic hormone were frequent in gonadectomised animals. Similar manifestations of lactogenic hormone secretion were much less common in old, normal animals bearing acidophil tumours.

### REFERENCES

BARRNETT, R. J., LADMAN, A. J. AND MCALLASTER, N. J.—(1955) *J. Histochem. Cytochem.*, **3**, 391.
BIELSCHOWSKY, F.—(1955) *Brit. J. Cancer*, **9**, 80.
CROSSMON, G.—(1937) *Anat. Rec.*, **69**, 33.
DICKIE, M. M. AND LANE, P. W.—(1956) *Cancer Res.*, **16**, 49.
GABE, M.—(1953) *Bull. Micr.*, appl., **3**, 152.
GOMORI, G.—(1950) *Amer. J. clin. Path.*, **20**, 665.
GRIESBACH, W. E. AND PURVES, H. D.—(1956) *Proc. Univ. Otago med. Sch.*, **34**, 1.
HALMI, N. S.—(1950) *Endocrinology*, **47**, 289.
HERLANT, M.—(1956) *Arch. Biol., Liège*, **67**, 89.
HOUSSAY, B. A., HOUSSAY, A. B., CARDEZA, A. AND PINTO, R. M.—(1955) *Schweiz med. Wschr.*, **85**, 291.
PURVES, H. D.—(1956) *Proc. R. Soc. Med.*, **49**, 1014.
*Idem* AND GRIESBACH, W. E.—(1951*a*) *Endocrinology*, **49**, 244.—(1951*b*) *Ibid.*, **49**, 427. —(1951*c*) *Ibid.*, **49**, 652.—(1954) *Ibid.*, **55**, 785.—(1955) *Ibid.*, **56**, 374.
WOOLLEY, G. W. AND DICKIE, M. M.—(1949) *Cancer Res.*, **9**, 373.
WOOLLEY, G. W.—(1958) *Ciba Colloquia on Endocrinology*, **12**, 122. London (J. & A. Churchill).